# Altered Resting State Functional Activity of Brain Regions in Neovascular Glaucoma: A Resting-State Functional Magnetic Resonance Imaging Study

**DOI:** 10.3389/fnins.2021.800466

**Published:** 2021-12-13

**Authors:** Chao Yu, Chu-Qi Li, Qian-Min Ge, Hui-Ye Shu, Xu-Lin Liao, Yi-Cong Pan, Jie-Li Wu, Ting Su, Li-Juan Zhang, Rong-Bin Liang, Yi Shao, Er-Ming Zeng

**Affiliations:** ^1^Department of Neurosurgery and Ophthalmology, The First Affiliated Hospital of Nanchang University, Nanchang, China; ^2^The First Clinical Medical College, Nanchang University, Nanchang, China; ^3^Department of Ophthalmology and Visual Sciences, The Chinese University of Hong Kong, Hong Kong, Hong Kong SAR, China; ^4^Department of Ophthalmology, Xiang’an Hospital of Xiamen University, Xiamen, China; ^5^Fujian Provincial Key Laboratory of Ophthalmology and Visual Science, Eye Institute of Xiamen University, Xiamen University School of Medicine, Xiamen, China; ^6^Department of Ophthalmology, Massachusetts Eye and Ear, Harvard Medical School, Boston, MA, United States

**Keywords:** neovascular glaucoma (NVG), percent amplitude of fluctuation (PerAF), resting-state functional MRI, anterior cingulate and paracingulate gyri, right superior occipital gyrus, superior frontal gyrus (orbital part), inferior temporal gyrus

## Abstract

**Background:** Neovascular glaucoma (NVG) is a serious eye disease that causes irreversible damage to the eye. It can significantly increase intraocular pressure and cause severe pain, as well as abnormal activity in the cortical and pre-cortical visual systems. However, there are few studies in this area. This trial assessed the altered regional brain activity in patients with NVG using the percentage of fluctuation amplitude (PerAF) method.

**Methods:** Resting-state functional MRI (rs-fMRI) scans were conducted in 18 individuals with NVG and 18 healthy controls (HCs), matched for education level, gender, and age. The PerAF method was applied to assess brain activity. Mean PerAF values of brain regions in NVG and HCs were compared using receiver operating characteristic (ROC) curves.

**Results:** Lower PerAF values were found in the NVG group than in controls in the right anterior cingulate and paracingulate gyri (ACG.R), right superior occipital gyrus (SOG.R) and left superior frontal gyrus (orbital part) (ORBsup.L) (*p* < 0.001). In contrast, PerAF value was higher in NVG patients than in controls in the left inferior temporal gyrus (ITG.L) (*p* < 0.001). The hospital anxiety and depression scale (HADS) and visual analog score (VAS) were significantly and positively correlated with PerAF in ITG.L (*r* = 0.9331, *p* < 0.0001; and *r* = 0.7816, *p* = 0.0001, respectively).

**Conclusion:** Abnormal activity in the patient’s brain regions further confirms that the NVG affects the entire brain, not just the visual pathways and posterior retinal mechanisms (including the hypothalamic lateral geniculate nucleus and the primary visual cortex). This strengthens our understanding of the NVG and provides potential diagnostic and therapeutic support for patients who are difficult to diagnose and treat early.

## Introduction

Neovascular glaucoma (NVG) is a secondary eye disease caused by a variety of ocular and systemic diseases and is closely related to retinal ischemia and hypoxia caused by diseases. Three of the most common causative conditions of NVG are ischemic central retinal vein occlusion (CRVO) (33%), diabetic retinopathy (DR) (33%), and ocular ischemic syndrome (13%) (Peng et al., 2021). Clinically, NVG can manifest as increased intraocular pressure, photophobia, severe eye pain, corneal swelling, and iris erythema, causing irreversible visual impairment and severely affecting the lives of patients worldwide. In Europe, NVG affects about 75,000–1,13,000 people per year, which is about 3.9% of the European population with glaucoma, while in Asia it occurs in a range between 0.7 and 5.1% of glaucoma patients (Mocanu et al., 2005; Kwon and Sung, 2017).

Although NVG has multiple etiologies, its clinicopathological development generally follows a specific course. When a patient has a disease that may cause NVG, it progresses from early glaucoma to open-angle glaucoma with corneal leakage, followed by corneal contraction and closure to become angle-closure glaucoma (Havens and Gulati, 2016). The development of NVG is often accompanied by unbearable pain, and advanced (stage III) NVG is not effectively treated with medications and often requires surgery, which commonly involves enucleation. Therefore, it is critical to diagnose NVG at an early stage and to provide patients with prompt and effective treatment. This study focused on stage III NVG and assessed patients altered functional activity of brain regions using PerAF.

Resting-state functional magnetic resonance imaging (rs-fMRI) is a non-invasive and convenient method that is widely used to assess regional brain activity. With the development of imaging technology, amplitude of low frequency fluctuation (ALFF) has been applied in various diseases such as major depressive disorder (Liu et al., 2014) and mesial temporal lobe epilepsy (Zhang et al., 2010). However, ALFF is sensitive to the scale of the raw signal, and the blood oxygenation level dependent (BOLD) units are arbitrary. A standard procedure is therefore used to divide the ALFF of each voxel by the global mean ALFF, called mALFF. Fractional ALFF (fALFF) provides a possible solution, but combines fluctuations across the frequency spectrum, so is not able to reveal amplitude characteristics at specific frequencies (Jia et al., 2020). Percentage amplitude of fluctuation (PerAF) here is proposed as a percentage signal definition borrowed from fMRI. PerAF is a scale-independent method that avoids confounding from voxel-specific fluctuation amplitudes in fALFF. Therefore, PerAF seems to be a valid, reliable and intuitive measure of voxel-level spontaneous BOLD activity (Jia et al., 2020). Currently, PerAF has been applied in studies of mild cognitive impairment (Yu et al., 2019), sleep deprivation (Zeng et al., 2020), retinal detachment (Yang et al., 2021) and Familial Cortical Myoclonic Tremor (with epilepsy type 1) (Wang et al., 2020). Although studies using PerAF are rare at present, given its advantages, it seems likely that its use will increase over time.

In the present study, we used PerAF to observe activity in the brain regions of patients with NVG. To our knowledge, this is the first research on NVG using PerAF, and we hypothesize that, as in similar studies using ALFF, abnormal activity will be observed using PerAF. This may help us to further understand the pathogenesis and effects of NVG, such as the rationale and potential effects of intense pain.

## Materials and Methods

### Subjects

**FIGURE 1:**
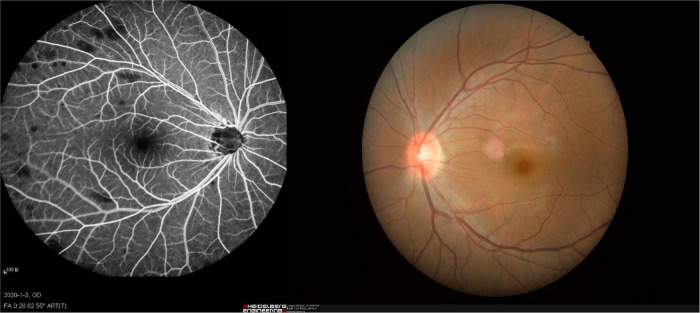
An example of NVG. NVG, neovascular glaucoma.

**FIGURE 2:**
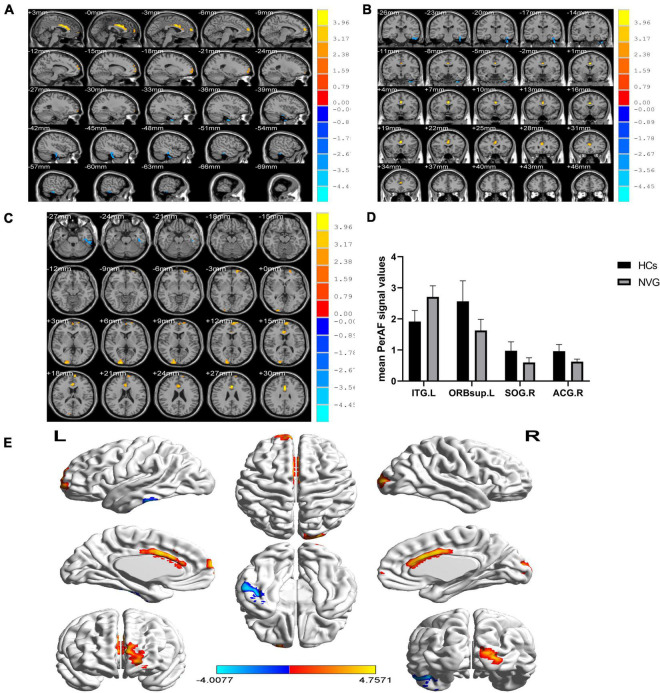
Relevant brain regions with statistical differences between healthy controls (HCs) and NVG in terms of PerAF **(A–E)**. Blue indicates lower PerAF values, while red indicates higher values. The histogram shows that the PerAF of ITG.L was significantly higher in the NVG group compared with the HCs, while the PerAF values of three brain regions, ORBsup.L, SOG.R, and ACG.R, were lower in NVG than controls. **(D)**. NVG, neovascular glaucoma; HCs, healthy controls; PerAF, percent amplitude of fluctuation; ITG.L, left inferior temporal gyrus; ORBsup.L, left superior frontal gyrus (orbital part); SOG.R, right superior occipital gyrus; and ACG.R, right anterior cingulate and paracingulate gyri.

Patients with NVG were recruited at the first affiliated hospital of Nanchang University, Department of ophthalmology. Patients satisfying the following criteria were eligible for inclusion: (1) presence of iris neovascularization (Figure 1); (2) clinical diagnosis of stage III NVG; (3) binocular involvement; (4) age over 40; and (5) all habitually right-handed. Exclusion criteria: (1) history of previous eye surgery; (2) diagnosis of cardiovascular, psychiatric or other systemic disease.

The following exclusion criteria applied to healthy controls enrolled in this study: (1) history of occupational disease (2) present diagnosis of cardiovascular disease or mental illness, (3) parenchymal malformation of the brain, or (4) contraindication to MRI scanning (such as pacemakers). During recruitment, the two groups were matched in age, handedness and sex.

The First Affiliated Hospital of Nanchang University, Medical Research Ethics Committee approved this study, which complied with the Declaration of Helsinki. Once the objectives, methods and possible risks of the study had been explained, all participants voluntarily cooperated and signed a declaration of informed consent.

### Parameters for Magnetic Resonance Imaging

Magnetic resonance imaging scans were conducted using a Trio 3 Tesla MRI scanner (Siemens, Munich, Germany). Each participant was instructed to keep eyes closed and to maintain stable breathing throughout the scan. To obtain whole-brain T1-weighted images, we modulated the corresponding metamorphic gradient echo sequence. The MRI parameters were set as follows: TE (echo time) = 2.26 ms, TR (repetition time) = 1,900 ms, gap = 0.5 mm, thickness = 1.0 mm, field of view (FOV) = 250 × 250 mm, acquisition matrix = 256 × 256, and rotation angle = 9°. The 3D metamorphic gradient echo plus series was applied to acquire images in the structural scanning session and the functional images were obtained with the following parameters: TE = 30 ms, TR = 2 000 ms, gap = 1.2 mm, thickness = 4.0 mm, FOV = 220 × 220 mm, acquisition matrix = 64 × 64, rotation angle = 90°, and 29 axial slices.

### Functional Magnetic Resonance Imaging Data Analyzing

To analyze the data obtained above, unusable data were filtered using MRIcro software, and the remaining data were processed using Data Processing Assistant for Resting-State fMRI (DPARSF V4.0) (Murta et al., 2015),^1^ and Statistical Parameter Mapping (SPM12 SPM).^2^ Making sure that patients have been fixated, the first ten volumes of measurement in each scan were removed to make data balanced. The DPARSF and SPM12 software were then used for head motion correction, slice timing, and spatial normalization. The data were smoothed using a full width half maximum Gaussian kernel of 6 mm × 6 mm × 6 mm. The fMRI scan data from individuals with head movement greater than 3 mm along the *x*-axis, *y*-axis, or *z*-axis and those from individuals with an angular range of more than 3 mm were excluded.

Higher-order models have been reported to help remove head motion effects, and for this purpose we used the Friston six head-motion parameters reported by Friston et al. (Liu et al., 2015). We also applied linear regression to remove other sources of artifacts, including signals from the regions of interest (ROI) to regions centered on the white matter of the brain (Fox et al., 2005). Finally, the fMRI images were processed using echo-planar image templates normalized to meet the spatial criteria of the Montreal Neurological Institute.

For each voxel, the PerAF value was calculated as follows:


(1) $$P\,e\,r\,A\,F=\frac{1}{n}\,\sum_{i=1}^{n}\left|\frac{x_{i}-\mu }{\mu }\right|\times 100\%$$

(2) $$\mu =\frac{1}{n}\,\sum_{i=1}^{n}x_{i}$$

where *X*_*i*_ shows signal intensity at the *i*_*th*_ time point, n represents the number of time points in the series, and μ refers to the mean of all values in the time series (Jia et al., 2020).

### Correlation Analysis

All patients with NVG completed the hospital anxiety and depression scale (HADS) questionnaire to measure if there complicates anxiety or depression, and a visual analog score (VAS). The best best-corrected visual acuity was derived applying logMAR test. Correlations between HADS and PerAF values and between VAS and PerAF at the four changed brain areas were determined using Pearson’s correlation analysis. Linear correlation plots were obtained using GraphPad Prism 9.0 software.

### Statistical Analysis

Using GraphPad prism 9, a two-sample *t*-test was performed to correct for the comparison of demographic and clinical performance between the HC and NVG groups. The two-sample *t*-test was also used in REST software to compare PerAF signal values between the two groups. Using Gaussian random field theory (GRF) for multiple comparisons, voxel-level threshold is 0.005, and cluster-level threshold is 0.05, and cluster value is 92. Receiver operating characteristic (ROC) curves were used to analyze and compare signal values extracted after multiple correction of brain regions between NVG and HCs. Pearson’s correlation analysis was applied test associations between regional PerAF signal values and behavioral data. In all analyses a *p*-value of < 0.05 was considered significant.

## Results

### Demographics and Characteristics

Eighteen patients with NVG and 18 HCs were recruited (8 men, 10 women in each group). No significant differences were found between the groups in age (*p* = 0.921), gender (*p* > 0.99), or handedness (*p* > 0.99). Significant differences (NVGs have a lower acuity) were found, however, in monocular best-corrected visual acuity (Left: *p* = 0.014; Right: *p* = 0.019) and intraocular pressure (Left: *p* = 0.009; Right: *p* = 0.013). More information is provided in Table 1.

**TABLE 1:** Demographics and behavioral results of neovascular glaucoma (NVG) and healthy controls (HCs) groups.

	NVG	HC	*t*-value	*p*-value
Male/female	8/10	8/10	N/A	>0.99
Age(years)	54.16 ± 10.26	53.95 ± 9.68	0.456	0.921
Handedness	18R	18 R	N/A	>0.99
Duration (ms)	24.56 ± 14.76	N/A	N/A	N/A
Best-corrected VA-L	0.25 ± 0.10	1.05 ± 0.10	5.732	0.014
Best-corrected VA-R	0.25 ± 0.15	0.95 ± 0.15	5.343	0.019
IOP-L	29.26 ± 11.21	14.67 ± 4.78	12.654	0.009
IOP-R	28.17 ± 1312	15.16 ± 3.33	10.512	0.013

*Independent t-tests comparing the age of two groups (P < 0.05) represented statistically significant differences). Male/female and Handedness were analyzed using chi-squared test. NVG, neovascular glaucoma; HCs, healthy controls; N/A, not applicable; VA, visual acuity; R, right; L, left; and IOP, intraocular pressure.*

### Percentage of Fluctuation Amplitude Differences

Compared with HCs, the PerAF values were significantly higher (*p* < 0.005, Table 2) in the left inferior temporal gyrus (ITG.L) of NVG patients. In contrast to this, values were lower in NVG than HCs in the right anterior cingulate and paracingulate gyri (ACG.R), left superior frontal gyrus (orbital part) (ORBsup.L) and right superior occipital gyrus (SOG.R) (Figure 2).

**TABLE 2:** Brain areas with significantly different PerAF values between the NVG and HCs.

Brain areas	MNI coordinates			BA	Number of voxels	*T* value
	X	Y	Z			
HC < NVG						
ITG.L	−36	−6	−51	55	107	−4.0077
HC > NVG						
ORBsup.L	−3	66	12	–	103	3.9027
SFG.R	18	−99	6	–	111	4.0601
ACG.R	3	18	27	–	155	4.7571

*The statistical threshold was set at a voxel level with P < 0.005 for multiple comparisons using Gaussian random field theory. PerAF, percent amplitude of fluctuation; BA, Brodmann area; HC, healthy control; MNI, Montreal Neurological Institute; NVG, Neovascular glaucoma; ITG.L, left inferior temporal gyrus; ORBsup.L, superior frontal gyrus (orbital part); SFG.R, right supraoccipital gyrus; and ACG.R, right anterior cingulate and paracingulate gyrus.*

### Receiver Operating Characteristic Curve

**FIGURE 3:**
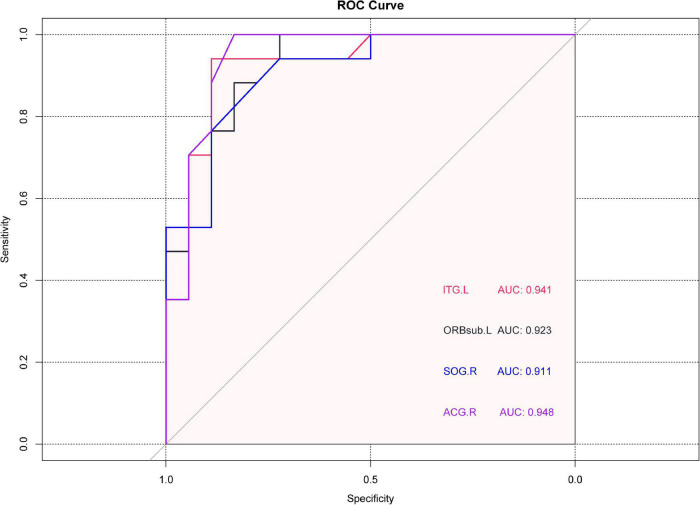
Receiver operating characteristic (ROC) curve analysis of the mean PerAF values brain regions showing abnormal values. The area under the ROC curve was 0.9414 (*p* < 0.0001, 95%CI: 0.8677–1.000) for ITG.L; 0.9228(*p* < 0.0001, 95%CI: 0.8362–1.000) for ORBsup.L; 0.9105 for SOG.R (*p* < 0.0001, 95%CI: 0.8178–1.000); 0.9475 for ACG.R (*p* < 0.0001, 95%CI: 0.8698–1.000). ROC, receiver operating characteristic; PerAF, percent amplitude of fluctuation; ITG.L, left inferior temporal gyrus; ORBsup.L, left superior frontal gyrus (orbital part); SOG.R, right superior occipital gyrus; and ACG.R, right anterior cingulate and paracingulate gyri.

In ROC analysis, the area under the curve (AUC) has a value of 0–1.0 and provides an indication of diagnostic accuracy, values closer to 1.0 indicating higher accuracy. In the ITG.L region, where PerAF activity was relatively high in the NVG group, the AUC was 0.9414 (*p* < 0.0001, 95%CI: 0.8677–1.000). In regions, with lower PerAF activity in NVG, AUC was as follows: ORBsup.L 0.9228 (*p* < 0.0001, 95%CI: 0.8362–1.000); SOG.R 0.9105 (*p* < 0.0001, 95%CI: 0.8178–1.000); ACG.R 0.9475 (*p* < 0.0001, 95%CI: 0.8698–1.000) (Figure 3).

### Correlation

**FIGURE 4:**
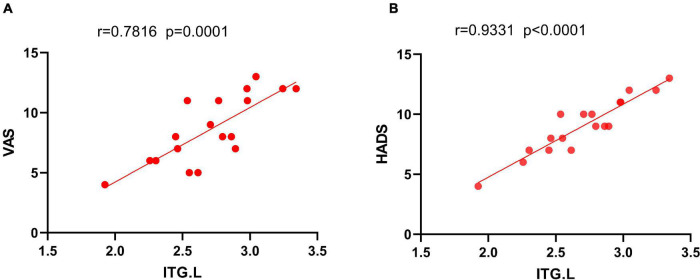
The correlation between the mean PerAF value of ITG.L and the VAS **(A)** and HADS **(B)**. In NVG group, the mean PerAF value of ITG.L showed a positive correlation with VAS (*r*^2^ = 0.7816, *P* = 0.0001). The mean PerAF value of ITG.L was also positively correlated with HADS (*r*^2^ = 0.9331, *P* < 0.0001). NVG, neovascular glaucoma; PerAF, percent amplitude of fluctuation; ITG.L, left inferior temporal gyrus; VAS, visual analog score; and HADS, the hospital anxiety and depression scale.

The HADS and VAS were significantly and positively correlated with PerAF in ITG.L (*r* = 0.9331, *p* < 0.0001; and *r* = 0.7816, *p* = 0.0001, respectively) (Figure 4).

## Discussion

**FIGURE 5:**
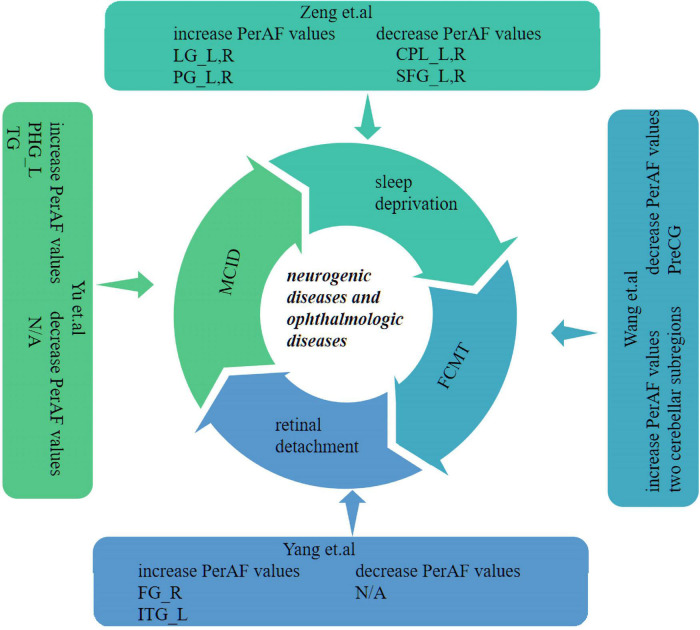
Previous studies which have applied the PerAF approach in neurogenic and ophthalmologic diseases. PerAF, percent amplitude of fluctuation; MCID, Mild cognitive impairment with depression symptom; L, left; R, right; LG, lingual gyrus; SFG, superior frontal gyrus; PG, post central gyrus; CPL, cerebellum posterior lobe; FG, fusiform gyrus; ITG, inferior temporal gyrus; TG, temporal gyrus; PHG, left para-hippocampus gyrus; FCMT, Familial Cortical Myoclonic Tremor (with epilepsy type 1); and PreCG, precentral gyrus.

**FIGURE 6:**
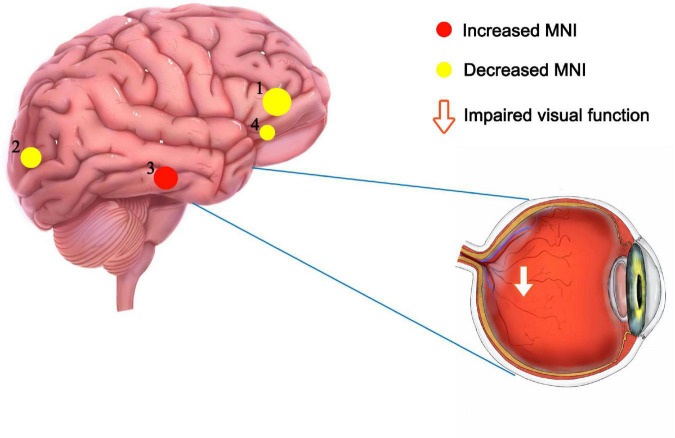
Significant differences in brain activity between the NVG and HCs. The differences were observed in the ITG.L, ORBsup.L, SOG.R, and ACG.R. The yellow means decreased PerAF brain regions, while the red denote increased PerAF brain regions. NVG, neovascular glaucoma; HCs, healthy controls; PerAF, percent amplitude of fluctuation; ITG.L, left inferior temporal gyrus; ORBsup.L, left superior frontal gyrus (orbital part); SOG.R, right superior occipital gyrus; and ACG.R, right anterior cingulate and paracingulate gyri.

Recent studies of neurological and ophthalmic diseases have shown abnormal PerAF values in disease-related brain regions (Figure 5). In the present study, this method was used to observe brain region activity in patients with stage III NVG. The study of Wu et al. (2020) showed abnormal intrinsic functional connectivity of the primary visual cortex in NVG patients, suggesting that NVG affects the brain. And we found an aberrant increased PerAF values of ITG.L and abnormally low PerAF values in brain regions of ORBsup.L, SOG.R, and ACG.R in patients with NVG compared to HCs, which may cause impaired visual function (Figure 6). The potential effects of alterations in various regions of the brain are discussed below and in Table 3.

**TABLE 3:** Altered brain regions and its potential impact.

Brain regions	Experimental result	Brain function	Anticipated results
Inferior temporal gyrus	HCs < NVG	Processing visual information; cognitive learning	Autism spectrum disorders; prosopagnosia; anxiety
superior frontal gyrus (orbital part)	HCs > NVG	Center for processing visual, spatial and emotional information	Emotional outbursts
Superior occipital gyrus	HCs > NVG	Visual cortical centers	Visual impairment predominant
Anterior cingulate and paracingulate gyri	HCs > NVG	Modulation of emotional responses; related to social behavior; impulse control	Depression; social anxiety; autism spectrum disorders

The inferior temporal gyrus (ITG) is one of the three temporal lobe gyri and is found in the lowermost part of the temporal lobe, connecting to the inferior occipital gyrus posteriorly and continuing down to the inferior surface of the temporal lobe. The ITG is one of the higher levels of visual processing, is involved in the representation of objects, faces and colors, and may also be involved in face perception. In a previous study, patients with autism had abnormally elevated ITG and fusiform fMRI values when undertaking a face recognition task (Schultz et al., 2000). Moreover, when NVG progresses to stage III, a series of symptoms such as vasoconstriction, peripheral conjunctival adhesions, as well as corneal swelling and congestion, severely damage the patient’s vision. In addition, an increase in intraocular pressure (IOP) is accompanied by severe eye pain (Peng et al., 2021). Thus, NVG patients have difficulty in visual recognition, which may lead to a compensatory increase in ITG activity, including ITG.L. VAS was used to evaluate the degree of eye pain. We analyze and access the correlation between VAS scores and PerAF values in this study, and found a positive correlation between VAS and PerAF values in ITG brain regions, but not other brain regions. These results indicate that more intense pain is associated with higher ITG.L activity in NVG patients. In addition to this, considering the mental impact of pain on patients, patients were asked to complete the HADS questionnaire and a positive linear correlation was found between this score and the PerAF value of ITG.L. This suggests that the mental status of NVG patients is also an important factor influencing ITG.L activity. Untreated, this could lead to other health disorders. According to Cai et al. (2018), patients with autism have increased ITG.L activity, the function of which includes recognition and socialization. In conjunction with the present results, the possibility exists for patients with NVG to develop a mental health, or psychiatric disorder, but there are no data to support this at this time.

The superior frontal gyrus (SFG) occupies approximately one-third of the frontal lobe. The function of the SFG is related to self-awareness, coordination of the sensory system (Goldberg et al., 2006), and stimulation of the SFG causes the subject to laugh and to feel joy and pleasure (Fried et al., 1998). The ORBsup.L is a part of the SFG. We suggest that the lower PerAF value of ORBsup.L in NVG than in the HC group may be related to severe ocular pain in NVG patients, which in turn leads to abnormally low PerAF values in ORBsup.L *via* nociception.

The main role of the occipital lobe is object recognition. The superior occipital gyrus is one of the three brain gyri of the occipital lobe, and is known as the occipital face area because together with the other two gyri of the occipital lobe, the inferior occipital gyrus and the middle occipital gyrus, it is involved in facial recognition (Albohn and Adams, 2016). According to Zijlstra et al. (2009), the SOG is involved in higher-level visual associative activity. Elevated intraocular pressure causes cortical and neurological damage to the brain in patients with glaucoma leading to a decrease in brain connectivity (Zhang et al., 2019). This may explain the reduced PerAF values of SOG.R observed in NVG patients in the present study, suggesting impairment of SOG.R. Reduced SOG activity may also be related to impaired visual acuity, along with reduced visual associative activity.

The cingulate cortex is situated medial to the cerebral cortex, usually regarded as a part of the limbic lobe, and is associated with the formative processing of emotions (Hadland et al., 2003), learning, and memory (Stanislav et al., 2013). The dorsal side of the anterior cingulate cortex (ACC), connected to the prefrontal cortex, is closely related to cognitive functions, while the ventral side, connected to the hippocampus, hypothalamus, and amygdala (Bush et al., 2000), is involved in moods, emotions, and other higher functions. MRI abnormalities in ACC are apparent in many diseases such as bipolar disorder (Fornito et al., 2008) and in the present study reduced ACG.R activity was found in NVG patients. This abnormality may reveal the neurological mechanisms underlying the pathogenesis of NVG and the possibility of mental health complications including anxiety and depression. In addition to this, ACC has been associated with pain perception. In very early studies (Foltz and White, 1962; Hurt and Ballantine, 1974), it was reported that surgical ablation of ACC and surrounding tissues could reduce pain (Johansen et al., 2001). One of the most important manifestations of stage III NVG is severe pain due to elevated IOP. Therefore, we suggest that the decreased PerAF of ACG.R in NVG patients also has a specific relationship with pain tolerance.

The present research has some limitations. First of all, this experiment is a small sample study. It limits the accuracy of the conclusion, does not rule out the possibility of bias, the need for more experiments to verify. Second, our target population was stage III NVG patients, so the results are not generalizable to NVG patients.

## Conclusion

The alterations in the four brain regions (ACG.R, SOG.R, ORBsup.L, ITG.L) described in this study in patients with NVG demonstrate that neuropathies in the NVG can affect the entire brain, not just the visual pathways and posterior retinal mechanisms (including the hypothalamic lateral geniculate nucleus and the primary visual cortex). And it may provide some new insights for diagnosis and treatment.

## Data Availability Statement

The raw data supporting the conclusions of this article will be made available by the authors, without undue reservation.

## Ethics Statement

The studies involving human participants were reviewed and approved by The First Affiliated Hospital of Nanchang University, Medical Research Ethics Committee. The patients/participants provided their written informed consent to participate in this study.

## Author Contributions

YS contributed to conception and design of the study. Q-MG and C-QL organized the database. CY performed the statistical analysis and wrote the first draft of the manuscript and sections of the manuscript. All authors contributed to manuscript revision, read, and approved the submitted version.

## Conflict of Interest

The authors declare that the research was conducted in the absence of any commercial or financial relationships that could be construed as a potential conflict of interest.

## Publisher’s Note

All claims expressed in this article are solely those of the authors and do not necessarily represent those of their affiliated organizations, or those of the publisher, the editors and the reviewers. Any product that may be evaluated in this article, or claim that may be made by its manufacturer, is not guaranteed or endorsed by the publisher.

## Footnotes

1
http://rfmri.org/DPARSF

2
https://www.fil.ion.ucl.ac.uk/spm/software/spm12/

## References

[B1] Albohn D. N. Adams R. B. Jr. (2016). “Social vision: at the intersection of vision and person perception,” in *Neuroimaging Personality, Social Cognition, and Character*, eds Absher J. R. Cloutier J. (Amsterdam: Elsevier), 159–186. 10.1186/s13229-018-0198-4

[B2] Bush G. Luu P. Posner M. I. (2000). Cognitive and emotional influences in anterior cingulate cortex. *Trends Cogn. Sci.* 4 215–222. 10.1016/s1364-6613(00)01483-2 10827444

[B3] Cai J. Hu X. Guo K. Yang P. Situ M. Huang Y. (2018). Increased left inferior temporal gyrus was found in both low function autism and high function autism. *Front. Psychiatry* 9:542. 10.3389/fpsyt.2018.00542 30425664 PMC6218606

[B4] Foltz E. L. White L. E. (1962). Pain “relief” by frontal cingulumotomy. *J. Neurosurg.* 19 89–100. 10.3171/jns.1962.19.2.0089 13893868

[B5] Fornito A. Malhi G. S. Lagopoulos J. Ivanovski B. Wood S. J. Saling M. M. (2008). Anatomical abnormalities of the anterior cingulate and paracingulate cortex in patients with bipolar I disorder. *Psychiatry Res. Neuroimaging* 162 123–132. 10.1016/j.pscychresns.2007.06.004 18207705

[B6] Fox M. D. Snyder A. Z. Vincent J. L. Corbetta M. Van Essen D. C. Raichle M. E. (2005). The human brain is intrinsically organized into dynamic, anticorrelated functional networks. *Proc. Natl. Acad. Sci. U.S.A.* 102 9673–9678. 10.1073/pnas.0504136102 15976020 PMC1157105

[B7] Fried I. Wilson C. L. MacDonald K. A. Behnke E. J. (1998). Electric current stimulates laughter. *Nature* 391 650–650. 10.1038/35536 9490408

[B8] Goldberg I. I. Harel M. Malach R. (2006). When the brain loses its self: prefrontal inactivation during sensorimotor processing. *Neuron* 50 329–339. 10.1016/j.neuron.2006.03.015 16630842

[B9] Hadland K. Rushworth M. F. Gaffan D. Passingham R. E. (2003). The effect of cingulate lesions on social behaviour and emotion. *Neuropsychologia* 41 919–931. 10.1016/s0028-3932(02)00325-1 12667528

[B10] Havens S. J. Gulati V. (2016). Neovascular glaucoma. *Dev. Ophthalmol.* 55 196–204. 10.1159/000431196 26501989 PMC5664957

[B11] Hurt R. W. Ballantine H. T. Jr. (1974). Stereotactic anterior cingulate lesions for persistent pain: a report on 68 cases. *Neurosurgery* 21(CN_Suppl. 1) 334–351. 10.1093/neurosurgery/21.cn_suppl_1.334 4370936

[B12] Jia X.-Z. Sun J.-W. Ji G.-J. Liao W. Lv Y. T. Wang J. (2020). Percent amplitude of fluctuation: a simple measure for resting-state fMRI signal at single voxel level. *PLoS One* 15:e0227021. 10.1371/journal.pone.0227021 31914167 PMC6948733

[B13] Johansen J. P. Fields H. L. Manning B. H. (2001). The affective component of pain in rodents: direct evidence for a contribution of the anterior cingulate cortex. *Proc. Natl. Acad. Sci. U.S.A.* 98 8077–8082. 10.1073/pnas.141218998 11416168 PMC35470

[B14] Kwon J. Sung K. R. (2017). Effect of preoperative intravitreal bevacizumab on the surgical outcome of neovascular glaucoma at different stages. *J. Ophthalmol.* 2017:7672485. 10.1155/2017/7672485 28713590 PMC5496122

[B15] Liu J. Ren L. Womer F. Y. Wang J. Fan G. Jiang W. (2014). Alterations in amplitude of low frequency fluctuation in treatment-naïve major depressive disorder measured with resting-state fMRI. *Hum. Brain Mapp.* 35 4979–4988. 10.1002/hbm.22526 24740815 PMC6869357

[B16] Liu X. Yan Z. Wang T. Yang X. Feng F. Fan L. (2015). Connectivity pattern differences bilaterally in the cerebellum posterior lobe in healthy subjects after normal sleep and sleep deprivation: a resting-state functional MRI study. *Neuropsychiatr. Dis. Treat.* 11 1279–1289. 10.2147/NDT.S84204 26064046 PMC4451850

[B17] Mocanu C. Barãscu D. Marinescu F. Lãcrãţeanu M. Iliuşi F. Simionescu C. (2005). Neovascular glaucoma–retrospective study. *Oftalmologia* 49 58–65. 16524128

[B18] Murta T. Leite M. Carmichael D. W. Figueiredo P. Lemieux L. (2015). Electrophysiological correlates of the BOLD signal for EEG-informed fMRI. *Hum. Brain Mapp.* 36 391–414. 25277370 10.1002/hbm.22623 PMC4280889

[B19] Peng Z. Y. Liu Y. X. Li B. Ge Q. M. Liang R. B. Li Q. Y. (2021). Altered spontaneous brain activity patterns in patients with neovascular glaucoma using amplitude of low-frequency fluctuations: a functional magnetic resonance imaging study. *Brain Behav.* 11:e02018. 10.1002/brb3.2018 33386699 PMC7994689

[B20] Schultz R. T. Gauthier I. Klin A. Fulbright R. K. Anderson A. W. Volkmar F. (2000). Abnormal ventral temporal cortical activity during face discrimination among individuals with autism and Asperger syndrome. *Arch. Gen. Psychiatry* 57 331–340. 10.1001/archpsyc.57.4.331 10768694

[B21] Stanislav K. Alexander V. Maria P. Evgenia N. Boris V. (2013). Anatomical characteristics of cingulate cortex and neuropsychological memory tests performance. *Procedia Soc. Behav. Sci.* 86 128–133. 10.1016/j.sbspro.2013.08.537

[B22] Wang B. Wang J. Cen Z. Wei W. Xie F. Chen Y. (2020). Altered cerebello-motor network in familial cortical myoclonic tremor with epilepsy type 1. *Mov. Disord.* 35 1012–1020. 10.1002/mds.28014 32129927

[B23] Wu Y.-Y. Wang S.-F. Zhu P.-W. Yuan Q. Shi W. Q. Lin Q. (2020). Altered intrinsic functional connectivity of the primary visual cortex in patients with neovascular glaucoma: a resting-state functional magnetic resonance imaging study. *Neuropsychiatr. Dis. Treat.* 16 25–33. 10.2147/NDT.S228606 32021196 PMC6954828

[B24] Yang Y. C. Li Q. Y. Chen M. J. Zhang L. J. Zhang M. Y. Pan Y. C. (2021). Investigation of changes in retinal detachment-related brain region activities and functions using the percent amplitude of fluctuation method: a resting-state functional magnetic resonance imaging study. *Neuropsychiatr. Dis. Treat.* 17 251–260. 10.2147/NDT.S292132 33536757 PMC7850567

[B25] Yu Y. Li Z. Lin Y. Yu J. Peng G. Zhang K. (2019). Depression affects intrinsic brain activity in patients with mild cognitive impairment. *Front. Neurosci.* 13:1333. 10.3389/fnins.2019.01333 31920500 PMC6928005

[B26] Zeng B. Zhou J. Li Z. Zhang H. Li Z. Yu P. (2020). Altered percent amplitude of fluctuation in healthy subjects after 36 h sleep deprivation. *Front. Neurol.* 11:565025. 10.3389/fneur.2020.565025 33519662 PMC7843545

[B27] Zhang Q. Shu Y. Li X. Xiong C. Li P. Pang Y. (2019). Resting-state functional magnetic resonance study of primary open-angle glaucoma based on voxelwise brain network degree centrality. *Neurosci. Lett.* 712:134500. 10.1016/j.neulet.2019.134500 31557522

[B28] Zhang Z. Lu G. Zhong Y. Tan Q. Chen H. Liao W. (2010). fMRI study of mesial temporal lobe epilepsy using amplitude of low-frequency fluctuation analysis. *Hum. Brain Mapp.* 31 1851–1861. 20225278 10.1002/hbm.20982 PMC6870704

[B29] Zijlstra F. Veltman D. J. Booij J. van den Brink W. Franken I. H. (2009). Neurobiological substrates of cue-elicited craving and anhedonia in recently abstinent opioid-dependent males. *Drug Alcohol Depend.* 99 183–192. 10.1016/j.drugalcdep.2008.07.012 18823721

